# Formulation of water pollutant discharge limits for malathion based on nonsensitive aquatic organism protection

**DOI:** 10.1038/s41598-023-43494-z

**Published:** 2023-10-12

**Authors:** Yuxia Wei, Qingqing Liu, Jian Zhao

**Affiliations:** State Key Laboratory of Environmental Criteria and Risk Assessment, Chinese Research Academy of Environmental Sciences, Beijing, 100012 China

**Keywords:** Environmental impact, Environmental sciences, Environmental social sciences

## Abstract

The “Integrated Wastewater Discharge Standard” was implemented for water pollutant discharge in China’s pesticide industry, which has no control requirements for particular wastewater pollutants in the industry. In the standard, certain pollutants discharge limits are limited strictly or loosely, resulting in practical management implementation difficulties. In view of the highly selective targeting characteristics of organic pesticide active ingredients in fungicides, insecticides, and herbicides, a method for deriving discharge limits based on the water quality criteria for pesticides for the protection of nonsensitive species is established based on the idea of fully protecting aquatic organisms beyond sensitive objects. Through the use of malathion as an example, by screening its toxicity data in different species of aquatic organisms, the sequence point with the most significant change in the acute toxicity sensitivity of the species is taken as the variation point in the cumulative frequency of the sensitive and nonsensitive species to derive the water quality criteria, using the species sensitivity distribution method as the scientific basis for determining the discharge limits. After a comparative analysis of different simulation models, the sigmoid model, with the best fit, is selected to determine that the sensitive species hazard concentration (HC_s_) of malathion to aquatic organisms in China is 46.4 µg/L, and the discharge limit derived from the HC_s_ based on the relationship between the environmental capacity and emissions is rounded to 250 µg/L. Studies showed that the relationship between the emissions limit derived from the water quality criteria for protecting nonsensitive species and malathion limit stipulated in the “Environmental Quality Standards for Surface Water” conforms to the corresponding relationship of the quality standard and discharge standard, which can be achieved by current pollution control technology, combined with water quality improvement. The discharge limit offers the advantages of technical accessibility and economic rationality.

## Introduction

Pesticides are an important means of production and widely used in agriculture, forestry, health, and other fields for controlling pests. China is a leading country in the production and use of pesticides, with the national pesticide output reaching 2.498 million tons in 2021. Pesticides can guarantee food security and agricultural product quality but also pose a threat to the ecological environment. Currently, enterprises manufacturing pesticides other than heterocyclic pesticides are subject to the “Integrated Wastewater Discharge Standard^1^” and provinces with local standards are subject to local pollutant discharge standards. The Ministry of Ecology and Environment is currently formulating a discharge standard for water pollutants for the pesticide industry and solicited public opinion for the second time in March 2022.

There are two common approaches in the regulation of wastewater discharges standard: technology-based and water-quality-based standards. The technology-based standards mainly reflect the level of pollution prevention and control technology requirements, and are difficult to combine with the water quality of specific watersheds. The water quality-based discharge standards has certain advantages in connecting with the goal of improving water environment quality, and are closely related to water environmental quality standards. Water quality criteria refer to the maximum acceptable dose (or harmless effect dose), concentration level, or pollutant limit in an aquatic ecological environment with certain natural characteristics with no harmful effects on specific objects (aquatic organisms or people). Water quality criteria are the scientific basis for deriving water quality standards and environmental quality standards and further formulating discharge standards. In recent years, with the increasing application of environment-friendly, efficient, low-toxicity, and low-residue pesticides, preliminary research found that the main varieties of pesticides in China have low toxicity to human health. However, all varieties of pesticides, such as insecticides, herbicides, and fungicides, are highly toxic to specific sensitive aquatic organisms. The technology-based discharge limits cannot effectively prevent and control environmental risks to aquatic organisms. Therefore, it is necessary to derive discharge limits based on water quality standards, and the establishment of water quality standards requires the derivation of water quality criteria. For this reason, the establishment of water quality criteria with the main goal of protecting aquatic organisms is the key to examining pesticide water quality standards. To further standardize the derivation of water quality criteria for freshwater organisms, the Ministry of Ecology and Environment issued the “Technical Guidelines for Deriving Water Quality Criteria for Freshwater Organisms” in 2022^2^. The Ministry of Ecology and Environment of China issued three freshwater organism water quality criteria, including for cadmium, ammonia nitrogen, and phenol, as well as nutrient criteria for lakes in the central and eastern lake regions in the country^3^. In recent years, at home and abroad systematically examined the theoretical methodology of water quality criteria and developed water quality criteria for different varieties of pollutants. On the basis of common methods implemented at home and abroad, it was determined that 95% of species must be protected in the derivation of the water quality criteria for aquatic organisms. However, living organisms are extremely sensitive to pesticide active ingredients. The criteria value and discharge limits derived based on the protection of 95% of species will be extremely strict and will not comply with the technical process for formulating discharge limits based on the principle of technical feasibility and will not be economically reasonable. Therefore, a method for determining discharge limits for pesticide active ingredients must be established to not only connect with protection objectives and water body functions but also offer economic and technical feasibility with a scientific basis.

Taking malathion, which is an organophosphorus pesticide, as an example, this research collects and screens 93 acute toxicity data points of plants, invertebrates, vertebrates, and other major biological species covering the aquatic ecosystem in China, involving 20 species, 16 families, and 5 phyla. On the basis of the species sensitivity distribution (SSD) curve, toxic concentration and cumulative frequency distribution curves are constructed using different models, such as logistic and sigmoid models. The concentration level for protecting nonsensitive species is derived to provide a reference basis for the formulation of limit values for characteristic pollutants, such as pesticide active ingredients, in the national discharge standard of the pesticide industry, which is currently being formulated, and provide scientific guidance for the formulation of local watershed water pollutant discharge limits based on the goal of water quality improvement.

## Establishment of derivation method for water quality criteria for protecting nonsensitive aquatic organisms

The SSD curve method is effective for the promotion of the effect data of individual species to the horizontal effect of the entire ecosystem and the mainstream method for the derivation of ecological environment water quality criteria in Europe, the United States, and other countries. This method uses a distribution model to construct the SSD curve by toxic concentration and cumulative probability. By analyzing toxicity data, it is proposed to determine the pollutant concentration that can protect most species in the ecosystem. Generally, the species hazard concentration (HC_s_), with a cumulative frequency of HC_5_, is used to determine the water quality criteria, that is, the concentration level at which 5% of species are endangered or 95% of species are protected. The criteria derivation method used in the United States posits that using 1% or 10% of the benchmark will result in overprotection or insufficient protection, so the statistically significant value of 5% is chosen as the protection level. The species sensitivity degree distribution method integrates the toxicity data of different species. Using this method, we can evaluate the protection level of affected species based on a specific proportion and determine the species category with the greatest risk. The EU’s water ecological criteria are similar to those of the United States, which also uses the SSD to establish a water quality criteria based on the protection of 95% of biological species from adverse impacts. In the protection criteria for aquatic organisms issued jointly by Australia and New Zealand, the four protection levels include protecting 99%, 95%, 90%, and 80% of species^4^. In the Netherlands, a 5% protection level is selected to derive the maximum allowable concentration, and 50% is selected to derive the highest risk concentration^5^.

Malathion is an efficient and highly selective organic phosphorus insecticide and acaricide with contact effects, gastric toxicity, and certain fumigation effects on living organisms. This study is based on the “Technical Guidelines for Deriving Water Quality Criteria for Freshwater Organisms^2^” and uses appropriate models to fit the SSD cumulative frequency. At the same time, this study uses a method for determining the cumulative frequency variation point for sensitive and nonsensitive species to deduce the criteria value for the protection of nonsensitive aquatic organisms after extrapolating the evaluation factors for the protection of nonsensitive species. Previous analyses of the acute toxicity data of tens of pesticide active ingredients indicated that the determination of the cumulative frequency variation point for sensitive and nonsensitive species abides by the following methods: (1) the cumulative frequency variation point is generally within the protection level range of 5–40%, that is, protecting at least 60% of species, and (2) finds the sequence point with the most significant change in the toxicity sensitivity data of species and across different species within the range (marked as Δ*S*_*i*_/*S*_*i*_, where *S*_*i*_ refers to the acute toxicity of the *i*th species index, and Δ*S*_*i*_ refers to the difference in the acute toxicity between the *i* + 1th species index and *i*th species index). If the maximum change in the toxicity sensitivity data is not cross-species, then the sub-high value will be used for the judging and so forth. (3) When a variation point does not exist across the different species within the range, then the minimum-protection-level upper limit of 60% is used as the sequence point. (4) The sequence point obtained in this study is taken as the cumulative frequency variation point for the sensitive and nonsensitive species to calculate the cumulative frequency and acute criteria value for nonsensitive species protection with the optimal fitting model.

## Derivation of water quality criteria for malathion for the protection of nonsensitive species

### Acquisition of acute toxicity data

In this study, the acute toxicity data of malathion to aquatic organisms are collected by searching the publicly available literature and existing toxicity databases, and the data of species that do not exist in China are eliminated. The toxicity data should meet the following conditions: the toxicity end point of the acute toxicity value (ATV) of algae should be EC_50_ (half inhibitory concentration), with an exposure time of 96 h; the ATV of animals should include LC_50_ (median lethal concentration) or EC_50_, with an exposure time of 48 and 96 h; and the acute value for the same effect (AVE) should be the geometric mean of all the data with the same receptor and the same exposure end point. The collected toxicity data cover at least three different trophic levels, including producers, and at least 10 species in the following biological groups: one type of cyprinidae fish in the order Cypriniformes, and one type of non-cyprinidae fish in the order Cypriniformes; one type of zooplankton; one type of non-fish benthic animal (e.g., shellfish, benthic crustaceans, and so on); one type of amphibian or other aquatic animal belonging to a phylum different from that of the aforementioned animals; and one type of phytoplankton or aquatic vascular plant. In addition, according to HJ 831, the derivation of the criteria for insecticides should include toxicity data for aquatic insects. In this study, the toxicity data for *Chironomides elongatus* are screened. The malathion acute toxicity data screening includes a total of 93 data points for 20 species, 16 families, and 5 phyla. The details are shown in Table 1.

**Table 1:** Acute toxicity of malathion to aquatic organisms.

Aquatic organisms	phyla	family	Latin name	End point	Acute value for the same effect/(µg/L)
Freshwater plants	Green Algae	Scenedesmus obliquus	*Scenedesmus obliquus*	96 h EC_50_	1690^6^
	Green Algae	Chlorella	*Pseudokirchneriella subcapitata*	96 h EC_50_	3801.8^7,8^
	Green Algae	Chlorella	*Chlorella vulgari*	96 h EC_50_	24870^7^
	Duckweed	angiosperma	*Spirodela polyrrhiza*	96 h EC_50_	13820^7^
	Duckweed	angiosperma	*Lemna minor*	96 h EC_50_	26120^7^
Invertebrates	Arthropoda	Daphniidae	*Daphnia magna*	48 h EC_50_	1.75^9,10^
	Arthropoda	Daphniidae	*Daphnia magna*	48 h LC_50_	7.28^11–14^
	Arthropoda	Daphniidae	*Ceriodaphnia dubia*	48 h EC_50_	0.84^15^
	Arthropoda	Daphniidae	*Ceriodaphnia dubia*	48 h LC_50_	1.55^16–18^
	Arthropoda	Daphniidae	*Ceriodaphnia dubia*	96 h LC_50_	1.91^19^
	Arthropoda	Daphniidae	*Daphnia pulex*	48 h EC_50_	1.90^20^
	Arthropoda	Mysidae	*Americamysis bahia*	96 h LC_50_	3.88^10,21^
	Arthropoda	Gammaridae	*Gammarus lacustris*	48 h LC_50_	1.80^10^
	Arthropoda	Gammaridae	*Gammarus lacustris*	96 h LC_50_	1.71^22^
	Arthropoda	Chironomidae	*Chironomus tentans*	48 h EC_50_	1.5^7^
	Mollusca	Oystidae	*Ostrea gigas tnunb*	96 h EC_50_	3008^10^
	Mollusca	Oystidae	*Ostrea gigas tnunb*	48 h EC_50_	9070^10^
Vertebrates	Chordates	Cyprinidae	*Danio rerio*	48 h LC_50_	1250^23^
	Chordates	Cyprinidae	*Danio rerio*	96 h LC_50_	403.4^23,24^
	Chordates	Ictaluridae	*Ictalurus punctatus*	48 h EC_50_	8900^25^
	Chordates	Ictaluridae	*Ictalurus punctatus*	48 h LC_50_	57300^26^
	Chordates	Ictaluridae	*Ictalurus punctatus*	96 h LC_50_	13,621.9^20,27^
	Chordates	Spinosauridae	*Lepomis macrochirus*	48 h EC_50_	86^25^
	Chordates	Spinosauridae	*Lepomis macrochirus*	48 h LC_50_	88^10^
	Chordates	Spinosauridae	*Lepomis macrochirus*	96 h LC_50_	75.2^10,20,28,29^
	Chordates	Cyprinidae	*Oryzias latipes*	96 h LC_50_	9700^30^
	Chordates	Cyprinidae	*Oryzias latipes*	48 h LC_50_	1800^31^
	Chordates	Poeciliidae	*Poecilia reticulata*	48 h LC_50_	1800^32^
	Chordates	Poeciliidae	*Poecilia reticulata*	96 h LC_50_	1928.7^32,33^
	Chordates	Salmonidae	*Salmo trutta*	96 h LC_50_	101^20^
	Chordates	Cyprinodontidae	*Cyprinodon variegatus*	96 h LC_50_	45.2^10^
	Chordates	Ranidae	*Rana chensinensis*	96 h LC_50_	1670^7^

### Fitting and evaluation of SSD curves

First, the AVE of each species and its logarithmic value lgAVE are calculated in Formula (1). Second, lgAVE is ranked from smallest to largest, and its rank R (the rank with the lowest toxicity value is 1, followed by rank 2, arranged in sequence. If two or more species have the same toxicity value, then they are arranged by any continuous rank) and the acute cumulative frequency F_R_ of each species are calculated separately. The calculation method is shown in Formula (2).

1 $$\text{AVE} = \sqrt[i]{{\text{ATV}_{1} \times \text{ATV}_{2} \times \ldots \times \text{ATV}_{\text{i}} }}$$

where AVE is acute value for the same effect, (μg/L or mg/L), ATV represents the acute toxicity value (μg/L or mg/L), and i represents a certain species.

2 $$\text{F}_{\text{R}}= \frac{R}{N + 1} \times 100\% ,$$

where FR is the cumulative frequency, R is the rank of the toxicity value (dimensionless), and N is the sum of all the frequencies (number).

With lgAVE as the independent variable x and the corresponding cumulative frequency FR as the dependent variable y, SSD model fitting is performed using the normal distribution model, logarithmic normal distribution model, logistic model, and logarithmic logistic model. The best fitting model is selected for the fitting, and the evaluation parameters include (a) the root mean square error (RMSE), and the closer the RMSE to 0, the higher the accuracy of the model fitting, and (b) the fitting correlation coefficient *R*^*2*^, and the larger the *R*^*2*^, the higher the correlation. The curve obtained from the optimal fitting model should match the data points participating in the fitting to ensure that the water quality criteria extrapolated from the fitted SSD curve are statistically reasonable and reliable.

**Figure 1:**
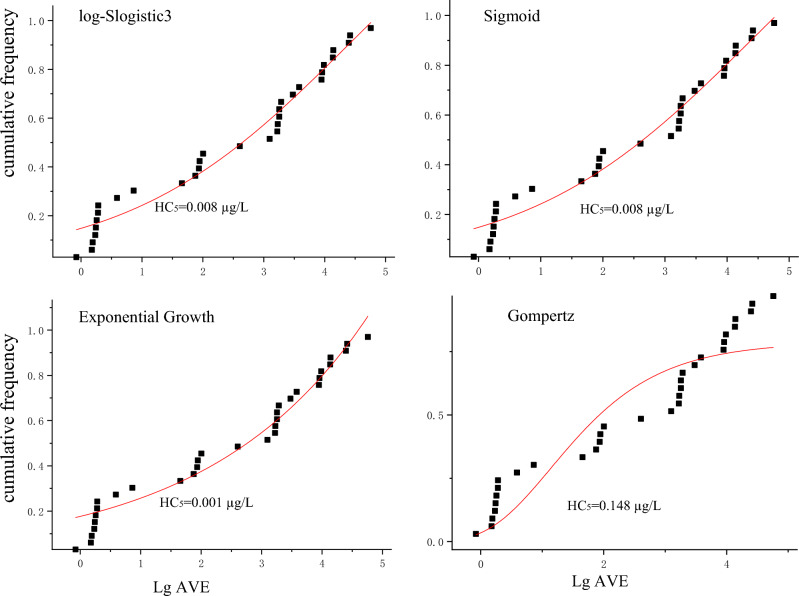
SSD curves of different models for acute toxicity data of malathion.

This study used Origin 2019 to draw the fitting curves and logistic, sigmoid, exponential growth, gompertz, and other models for the SSD fitting. The model fitting parameters are listed in Table 2, and the fitting curves are shown in Fig. 1. In the comparison of the different distribution models for deriving the water quality criteria, this study found that the fitting results of the log-slogistic model were close to those of the sigmoid model. The RMSE of the two models was the same, but the fitting correlation coefficient *R*^*2*^ of the sigmoid model was higher than that of the log-slogistic model. Thus, the sigmoid model was taken as the malathion HC_5_ derivation model, with an HC_5_ value of 0.008 µg/L.

**Table 2:** Fitting results of malathion using different models.

SSD model	Fitting formula	Parameters	RMSE	*R*^2^	HC_5_/(µg/L)
Log-slogistic	$y = a/(1 + b \times e^{ - kx} )$	*a* = 1.806, *b* = 11.158, *k* = 0.549	0.049	0.960	0.008
Sigmoid	${y = a/(1 + e}^{{{(k - x)/b}}})$	a = 1.804, b = 1.822, k = 4.394	0.049	0.969	0.008
Exponential growth	$y = a \times e^{bx}$	*a* = 0.176, *b* = 0.377	0.055	0.961	0.001
Gompertz	$y = a \times e^{ - e(k - x)/b}$	*a* = 0.788, *b* = 3.039, *k* = 2.252	0.108	0.850	0.148

### Determination of the cumulative frequency variation point for sensitive and nonsensitive species

**Figure 2:**
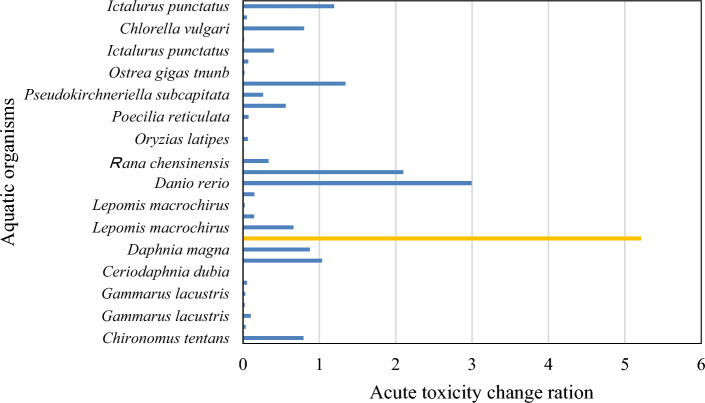
Sensitivity changes in acute toxicity data of malathion in aquatic organisms.

On the basis of the statistics of the 93 acute toxicity data points of malathion for invertebrates, vertebrates, and plants, covering 20 species, 16 families, and 5 phyla, the sample values were ranked from lowest to highest, and the acute toxicity change ratio (Δ*S*_*i*_/*S*_*i*_) was calculated. Figure 2 shows the results, with the aquatic biological species taken as the abscissa and the acute toxicity change ratio taken as the ordinate.

Within a protection level of 5–40% for aquatic organisms, the sequence point with the most significant change in the acute toxicity sensitivity data across the different species categories was identified. As shown in Fig. 2, the maximum sensitivity change (Δ*S*_*i*_/*S*_*i*_ = 5.21) in the acute toxicity data of malathion was observed in *variegated killifish*. Crustaceans such as *Daphnia magna* are located in front of *Variegated killifish*, whereas fish belonging to different phyla are located behind *Variegated killifish*. Therefore, the sequence of *Variegated killifish* was used as the split point of the cumulative frequency of the sensitive and nonsensitive aquatic organisms. Among them, the sequence of *Variegated killifish* was 11, and its corresponding cumulative frequency value was calculated to be 33.3%. On the basis of the sigmoid model, the protection concentration for the nonsensitive aquatic organisms was determined to be 46.4 µg/L, which meant that the malathion concentration for protecting 67% of the nonsensitive aquatic organisms was 46.4 µg/L. The HC_5_ and HC_s_ values of malathion were 0.008 µg/L and 46.4 µg/L, respectively.

### Derivation of water quality criteria value

The value of y is taken as the cumulative frequency value, and the corresponding x value is calculated. Then, the opposition number (10^x^) of x is used as the corresponding HC_5_ or HC_s_ value. According to Formula (3), the water quality criteria for malathion for aquatic organisms are deduced.

3 $$\text{SWQC} = \frac{SHC}{{SAF}},$$

where SWQC is the short-term water quality criteria for aquatic organisms (μg/L or mg/L), SHC represents the species hazard concentration derived from the acute toxicity data (μg/L or mg/L), and SAF is the assessment factor for the short-term water quality criteria for aquatic organisms (dimensionless).

The SAF value is determined comprehensively based on the number of data points used to derive the criteria, coverage range of the tested species, and data fitting distribution. The general value is 2–5 when the number of species included in the effective toxicity data is greater than 15, and the SAF value is 2. In this study, the SAF value is 2. According to Formula (2), the acute water quality criteria value for the full protection of 95% of aquatic organisms by malathion was 0.004 µg/L, and the acute criteria value for the protection of nonsensitive aquatic organisms was 23.2 µg/L.

## Derivation of discharge limits of malathion

Water quality criteria are the basis for formulating water environmental quality standards. After the establishment of water quality criteria, conducting research on the transformation of the water quality criteria into water environmental quality standards and emissions standards is necessary to effectively provide technical support for environmental management. The formulation of the discharge limits based on the water quality criteria value requires the comprehensive consideration of various influencing factors, including the hydrological conditions of the receiving water, water body objectives and functions, the quantitative relationship between the discharge volume and water quality, the environmental management level and demand, the discharge limits of particular pollutants in existing emissions standards, and the economic and technological feasibility of reaching discharge limits. The relationship between pollutant discharge from outfall and the water environment quality was mainly considered in this research. The dilution multiple method was adopted, and the dilution multiple was 10 times, which is lower than the maximum dilution multiple of 20 times in the “Technical Guideline for the Development of National Water Pollutant Discharge Standards^34^”. Technical and economic accessibility in the pesticide industry was also considered to reach this limit. The discharge limit derived from the acute criteria value for the protection of the nonsensitive aquatic organisms was 232 µg/L, rounded to 250 µg/L. The discharge limit derived from the acute criteria value for the protection of 95% of the aquatic organisms was 0.04 µg/L.

## Evaluation of discharge limits of malathion

### Comparison of discharge limits of malathion at home and abroad

**Figure 3:**
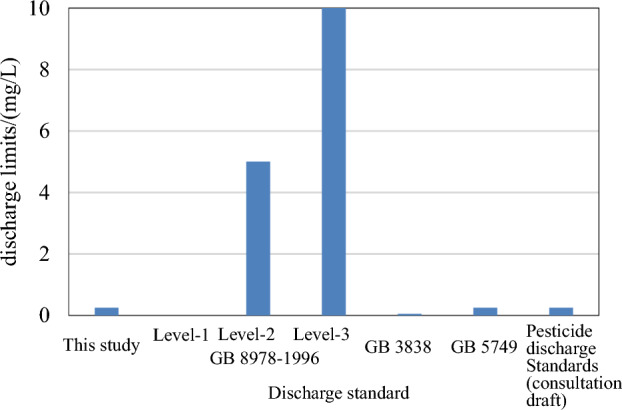
Comparison between malathion discharge limits in this study and those in relevant standards in China.

The discharge limit of malathion deduced from this study was 0.25 mg/L and compared with the level-1 limit, level-2 limit, and level-3 limit in the “Integrated Wastewater Discharge Standard” (GB 8978–1996)^1^; centralized drinking water limits in the “Environmental Quality Standard for Surface Water” (GB 3838–2002)^10,35^; and limits in the “Standards for Drinking Water Quality” (GB 5749–2022)^36^ in Fig. 3(SAMR, 2022).The level-1 limit in GB 8978 was “not detectable.” As the sensitivity of instruments is constantly improving, method detection limits have decreased. In terms of discharge requirements for malathion production enterprises, the “not detectable” status does not match the actual situation. However, the level-2 and level-3 limits were 5 mg/L and 10 mg/L, respectively, but not adequately strict and significantly less strict than the discharge limits deduced by the nonsensitive water quality criteria. The discharge limit deduced from this study was close to that in GB 5749 and discharge standard for the pesticide industry (consultation draft), slightly looser than the standard limit of centralized water sources, which conforms to the corresponding relationship between the quality and discharge limit in HJ 945.2–2018.

In China's local water pollutant discharge standards, some provinces, such as Beijing, Shanghai, Tianjin, etc., have stipulated emission limits for malathion. Beijing requires that wastewater discharged into Class II and III water bodies comply with the A discharge limit of 0.05 mg/L, and wastewater discharged into Class IV and V water bodies comply with the B discharge limit of 0.05 mg/L(DB11/307–2013)^37^. Shanghai requires the pollutant discharging units that directly discharge water pollutants into sensitive waters to implement the first level standard limit, and the pollutant discharging units that directly discharge water pollutants into non sensitive waters to implement the second level standard, where the first level limit of malathion is "not detectable", and the second level limit is 5.0 mg/L (DB31/199–2018)^38^. Tianjin and Shanghai have similar requirements for malathion (DB12/356–2018)^39^.

The malathion discharge limits deduced from this study were compared with the emissions standard limits of foreign pesticide industries. The US “Effluent Limitations Guideline, Pretreatment Standards, and New Source Performance Standards for the Pesticide Chemicals Manufacturing Point Source Category” stipulated new source performance standards, the existing source performance standards, and pretreatment standards for 91 pesticide active ingredients^40^. However, the limits are the load standard value of pollutant emissions per unit product. For example, the daily maximum malathion emissions of existing sources are 2.35 × 10^−7^ ton of pollutant per 1 ton product. In line with the wastewater discharge in the China Second National Pollution Source Survey (8.53 t/t product), the value was converted to 0.03 mg/L. According to the World Bank’s “Environmental, Health, and Safety Manual for Pesticide Industry^41^”, the discharge limit of pesticide active ingredients should be 0.05 mg/L. In 2011, India issued and implemented emissions regulations for the pesticide industry, in which the discharge limit of malathion and other active ingredients was set to 0.1 mg/L^42^. The discharge limit of malathion in the abovementioned countries or organizations is basically in the same order of magnitude as the discharge limits derived in this study, which is slightly looser than that in developed countries, with a certain rationality.

### Evaluation of applicability of discharge limits of malathion

The annual output of malathion in China is less than 10,000 tons. Major malathion production enterprises are distributed mainly in Shandong, Liaoning, Hebei, and other provinces. The wastewater produced in the production process mainly contains high concentrations of organic substances. Presently, pretreatment technology and biochemical treatment technology are adopted in China. Pretreatment technology mainly involves adsorption, extraction, advanced oxidation, and so on. After treatment, the organic matter content of wastewater can be reduced, and the biodegradability of wastewater can be improved. Then, the pretreated wastewater can be mixed with low-concentration wastewater, such as that in utilities, for biochemical treatment. For example, the ultrasonic-electrolysis-biochemical method is used to degrade malathion wastewater at Hebei University, which reached a total phosphorus degradation rate of more than 90% and COD_Cr_ degradation rate of more than 98%^43^. Mohammad examined the feasibility of MWCNTs for adsorbing and removing malathion, which is an organophosphorus pesticide, from water and the effects of the amount of adsorbent, reaction time, temperature, and other parameters on the removal of malathion^44^. Under certain conditions, MWCNTs can effectively remove nearly 100% of malathion from water. In addition, wet oxidation technology has gradually attracted the attention of pesticide enterprises in the treatment of hardly degradable pesticide wastewater, as it can effectively improve the biodegradability of wastewater and remove organic phosphorus pesticide wastewater containing malathion, dimethoate, and so on. After treatment with the aforementioned techniques, malathion concentrations in wastewater can reach the limits derived from the acute criteria value of the nonsensitive aquatic organisms. The method offers the merit of accessibility but differs from the method in which the value derived from the acute criteria value for full protection is 95% of aquatic organisms.

## Conclusions

Through the collection and screening of 93 acute toxicity data points in 20 species, 16 families, and 5 phyla, covering fish and zooplankton, benthos, phytoplankton, and amphibians, the acute water quality criteria value of malathion was derived by SSD fitting with the best model, that is, the sigmoid model, after evaluation with different fitting models.

The water quality criteria values obtained by extrapolating the HC_5_ and HC_s_ values represented different levels of protection for aquatic organisms, and the HC_s_ reflected the protection requirements for pesticide active ingredients for nonsensitive species. Given the high selectivity of pesticide active ingredients and high sensitivity of organisms, establishing a strict derivation method for the protection of nonsensitive species under the criteria value condition of protecting 95% of aquatic organisms is reasonable and scientific, which is not only in line with the actual emissions level achieved by enterprises using the best available technology but also linked with the quality of the water environment within a certain range.

## Data Availability

All data generated or analysed during this study are included in this published article.
